# The Hitchhiker's Guide to Virotherapy

**DOI:** 10.18632/oncotarget.627

**Published:** 2012-08-19

**Authors:** Oliver Donnelly, Richard Vile, Hardev Pandha, Kevin Harrington, Alan Melcher

**Affiliations:** Leeds Institute of Molecular Medicine, St. James's University Hospital, Leeds, UK; Molecular Medicine Program and Department of Immunology, Mayo Clinic, Rochester, MN, USA; Postgraduate Medical School, University of Surrey, Guildford, UK; Institute of Cancer Research, Centre for Cell and Molecular Biology, Chester Beatty Laboratories, London, UK; Leeds Institute of Molecular Medicine, St. James's University Hospital, Leeds, UK

In contrast to most other cancer therapies, early research with oncolytic viruses (OV) tended to use direct or intratumoural routes of administration, initially motivated by concerns that the major limitation of intravenous delivery would be immune attack against the viruses, whether by complement, cytokine or most critically neutralising antibodies. Even in the absence of preformed circulating neutralising antibodies, antiviral immunity develops rapidly over the course of multiple viral administrations [1]. Preclinically ex vivo cell preparations have been used to load virus onto or into assorted cells, including white blood cells (often called ‘hitchhiking’), but such manoeuvres would be difficult to implement clinically.

Given these perceived limitations, many have chosen direct administration to maximise intratumoural delivery: H101 (an oncolytic adenovirus with selectivity against p53-defective tumour cells) is approved in China and is given intratumourally for head and neck cancers. Several viruses have been administered into the tumour bed perioperatively for glioma. Measles has been administered intraperitoneally in ovarian cancer. One of the field-leading products is T-Vec (aka OncoVex), an engineered herpes virus. Its recent acquisition by Amgen was a fillip for those in the OV field, and was based on promising phase II data in melanoma where the virus was injected intratumourally into all accessible lesions [2]; a phase III study has closed to recruitment and results are keenly awaited. However these direct delivery routes are unpleasant for patients, unpopular with clinicians and unachievable for many deep-seated tumours [3].

We previously recovered replication-competent reovirus from tumour following intravenous infusion in three patients with head and neck cancer, [4] a finding recently replicated by Breitbach and colleagues using JX-594, an engineered vaccinia derivative [5]. Following intravenous injection JX594 was detected in whole blood by quantitative polymerase chain reaction (qPCR) for at least four hours after infusion. Patients underwent tumour biopsies 8-10 days after viral administration, and these biopsies were examined by PCR and by immunohistochemistry (IHC) for native viral protein and expression of the engineered marker gene. Amongst the eight subjects treated in the higher dose cohorts, all eight had at least one positive viral marker; four were positive across all three assays.

These vital observations that two different viruses, administered intravenously, reach tumour tissues strongly support the further development of OV for systemic use in metastatic disease. However, neither of these studies completely addressed the issue of how a systemically administered virus might successfully run the gauntlet of anti-viral immunity.

Reovirus is an endemic human pathogen and, therefore, neutralising anti-reovirus antibodies (NARA) are extremely prevalent in the population. In contrast vaccinia is not present in the environment and routine vaccination against smallpox ended in North America in the mid-1970s. In fact only one of the subjects treated with high-dose JX-594, in whom virus was detectable in the tumour, had pre-existing neutralising anti-vaccinia antibodies. In addition it is highly likely that successful virotherapy will require several doses of OV, which will induce high titres of neutralising antibodies in most patients.

Against this background, we have recently confirmed successful intravenous delivery of reovirus in a population of pre-immune cancer patients [6]. In a phase Ib study we administered intravenous reovirus to 10 patients prior to surgical resection of colorectal hepatic metastases. Patients received up to 5 daily infusions of reovirus, 6-28 days before surgery. Cell suspensions prepared from a number of tumours yielded replicating reovirus, but adjacent normal liver tissue did not. IHC confirmed these results and provided evidence of selective reovirus replication within tumours.

Importantly, all patients in the study had detectable neutralising antibodies before treatment and NARA titres peaked in all patients by the time of surgery. Accordingly, although reovirus was detected by PCR in the plasma of patients shortly after infusion, we were unable to recover replicating virus from plasma. This finding is compatible with NARA functionally neutralising the virus in the circulation. However we recovered replication-competent virus from the peripheral blood mononuclear cell, granulocyte and platelet fractions of the patients’ blood. These data indicate that virus hitchhikes on these circulating blood cells, circumventing the anti-viral immune response to access tumour. Hitchhiking virus onto *ex vivo*-cultured carrier cells is an area that we and others have explored preclinically [7,8], but had never been addressed in patients before. Remarkably our results demonstrate that this phenomenon occurs *in vivo* following intravenous administration of neat virus. Further work to characterise and enhance the mechanism of viral carriage is underway, and will be critical to optimising systemic virotherapy.

It is an exciting time to be involved in OV research. Preclinical developments continue to inform the clinical approach and provide novel targeted or armed agents for therapeutic testing [9]. Clinical studies have moved from addressing initial concerns about the safety of patients (and their attendants) to examining viral efficacy; thankfully there are reassuring data to address both questions. Understandable concerns about the fate of intravenously administered OV led to an initial preponderance of clinical trials examining direct routes of administration, but the three studies outlined above demonstrate that the intravenous delivery of OV is achievable, and anti-viral immunity is surmountable. We expect this will inform the next tranche of trials, as OV hopefully move from experimental therapies to mainstream cancer treatments.
